# A Comparison of RNA-Seq Results from Paired Formalin-Fixed Paraffin-Embedded and Fresh-Frozen Glioblastoma Tissue Samples

**DOI:** 10.1371/journal.pone.0170632

**Published:** 2017-01-25

**Authors:** Anna Esteve-Codina, Oriol Arpi, Maria Martinez-García, Estela Pineda, Mar Mallo, Marta Gut, Cristina Carrato, Anna Rovira, Raquel Lopez, Avelina Tortosa, Marc Dabad, Sonia Del Barco, Simon Heath, Silvia Bagué, Teresa Ribalta, Francesc Alameda, Nuria de la Iglesia, Carmen Balaña, on behalf of the GLIOCAT Group

**Affiliations:** 1 CNAG-CRG,Centre for Genomic Regulation (CRG), Institute of Science and Technology (BIST), Universitat Pompeu Fabra (UPF), Barcelona, Spain; 2 Cancer Research Program, Institut Hospital del Mar d’Investigacions Mèdiques (IMIM), Barcelona, Spain; 3 Medical Oncology, Hospital del Mar, Barcelona, Spain; 4 Medical Oncology, Hospital Clínic, Barcelona, Spain; 5 Institut de Recerca Contra la Leucèmia Josep Carreras, Badalona, Barcelona, Spain; 6 Sequencing Unit, CNAG-CRG, Centre for Genomic Regulation (CRG), Barcelona Institute of Science and Technology (BIST), Universitat Pompeu Fabra (UPF), Barcelona, Spain; 7 Pathology Department, Hospital Universitari Germans Trias i Pujol, Badalona, Barcelona, Spain; 8 Pathology Department, Hospital Josep Trueta, Girona, Spain; 9 Laboratori de quimio-resistència i cáncer, Facultat de Medicina i Ciències de la Salut, Universitat de Barcelona, Departament Infermeria Fonamental, Institut d'Investigacio Biomedica de Bellvitge (IDIBELL), Bellvitge, Barcelona, Spain; 10 Medical Oncology, Institut Català Oncologia (ICO), Hospital Josep Trueta, Girona, Spain; 11 Pathology Department, Hospital de Sant Pau, Barcelona, Spain; 12 Pathology Department (Neuropathology), Hospital Clinic, Barcelona, Spain; 13 Pathology Department, Hospital del Mar, Barcelona, Spain; 14 Glioma and Neural Stem Cell Group, Translational Genomics and Targeted Therapeutics in Solid Tumors Team, August Pi i Sunyer Biomedical Research Institute (IDIBAPS), Barcelona, Spain; 15 Institut Català d’Oncologia, Medical Oncology Service, Hospital Germans Trias i Pujol, Badalona, Spain; University of Navarra, SPAIN

## Abstract

The molecular classification of glioblastoma (GBM) based on gene expression might better explain outcome and response to treatment than clinical factors. Whole transcriptome sequencing using next-generation sequencing platforms is rapidly becoming accepted as a tool for measuring gene expression for both research and clinical use. Fresh frozen (FF) tissue specimens of GBM are difficult to obtain since tumor tissue obtained at surgery is often scarce and necrotic and diagnosis is prioritized over freezing. After diagnosis, leftover tissue is usually stored as formalin-fixed paraffin-embedded (FFPE) tissue. However, RNA from FFPE tissues is usually degraded, which could hamper gene expression analysis. We compared RNA-Seq data obtained from matched pairs of FF and FFPE GBM specimens. Only three FFPE out of eleven FFPE-FF matched samples yielded informative results. Several quality-control measurements showed that RNA from FFPE samples was highly degraded but maintained transcriptomic similarities to RNA from FF samples. Certain issues regarding mutation analysis and subtype prediction were detected. Nevertheless, our results suggest that RNA-Seq of FFPE GBM specimens provides reliable gene expression data that can be used in molecular studies of GBM if the RNA is sufficiently preserved.

## Introduction

Genomic profiling studies of glioblastoma (GBM) have established that GBM can be sub-classified into different intrinsic subtypes according to gene expression. Molecular classifications of GBM might better explain differences in outcome and response to treatment rather than morphological or clinical factors [1–3]. Gene expression studies have traditionally been performed using RNA extracted from fresh-frozen (FF) tissue. However, the availability of FF GBM tumour samples is very low as tumor tissue obtained from surgery is often scarce and necrotic. Moreover, the preservation of FF tissue is usually hampered by the priority task of obtaining a pathological diagnosis, performing an immunohistochemical study, and assessing O6-methylguanine-DNA methyltransferase (*MGMT*) promoter methylation status. Residual tissue, if existing, is routinely stored as formalin-fixed paraffin-embedded tissue (FFPE). Therefore, FFPE tissues represent an exploitable source of tumour material that can be used to perform the molecular studies in relation to clinicopathological information and known prognostic factors that are especially valuable in low-incidence diseases like GBM. RNA extracted from archival FFPE tissues has often suffered chemical modification, cross-linking, and degradation over time as a result of the fixation and archiving methods. Nevertheless, FFPE RNA has been successfully extracted from stored specimens [4, 5] and used for next-generation sequencing with successful results [6, 7]. Recent reports have demonstrated the feasibility of RNA-Seq in FFPE samples of several solid tumours, including glioblastoma [8–17]. However, whether the information gathered from RNA-Seq in FFPE GBM tissues is similar to that obtained from FF samples is still an open question.

We have performed a pilot study to determine whether gene expression data obtained from FFPE GBM tumour samples was comparable to that obtained from paired FF samples from the same tumour when assessed by RNA-Seq using the Illumina platform.

## Materials and Methods

### Patients and samples

This study was approved by the Institutional Review Board of the Hospital Germans Trias i Pujol (PI-14-016) and by the Ethics Committees of all the participating institutions and conducted in accordance with the Declaration of Helsinki.

We selected eleven cases from a database of 432 GBM patients for whom we had both FFPE and FF tumour samples. All patients had primary glioblastomas, as confirmed by pathological review (FA, SB, CC, TR, RL). Two samples had been obtained from the same tumour from each patient, one of which had been stored as FFPE and one as FF.

### RNA extraction and assessment of quality

The RNA extraction of FF and FFPE tumor specimens was performed on five 15μm-deep tissue cuts using the RNeasy Mini Kit (Qiagen), according to the manufacturer’s recommendations. RNA quantity and purity were measured with the NanoDrop ND-1000 spectrophotometer (Thermo Scientific). RNA integrity, determined by the RNA integrity number (RIN), was determined with the 2100 Bioanalyzer (Agilent).

### RNA library construction and sequencing

Samples were sequenced at Centro Nacional de Análisis Genómico (CNAG-CRG, Barcelona, Spain). A modified TruSeq™ Stranded Total RNA kit protocol (Illumina Inc.) was used to prepare the RNA-Seq libraries from FFPE samples. Ribosomal RNA (rRNA) was depleted from 0.5–1.0 ug of total RNA using the RiboZero Magnetic Gold Kit (Human/Mouse/Rat, Epicentre). rRNA-depleted RNA samples were purified using Agencourt RNA Clean XP beads (Beckman Coulter Genomics) and RNA was eluted with the Elute, Prime, Fragment Mix from the TruSeq Stranded Total RNA kit. The RNA fragmentation time was shortened to 2.5 minutes due to the low quality of the initial total RNA (assessed by Eukaryote Total RNA Nano Bioanalyzer assay, Agilent). Following the fragmentation, first and second strand synthesis, the Illumina bar-coded adapters were ligated at 1/10 dilution of the recommended concentration. Libraries were enriched with 15 cycles of PCR. The size and quality of the libraries were assessed in a High Sensitivity DNA Bioanalyzer assay (Agilent).

The starting input material for the libraries construction was DNA free total RNA from FF using the TruSeq™ Stranded Total RNA kit protocol (Illumina Inc.), according to the manufacturer’s protocol with some modifications for the FFPE samples, and the final library was quality controlled in Agilent DNA 7500 Bioanalyzer assay (Agilent).

Each library was sequenced using TruSeq SBS Kit v3-HS (Illumina), in paired-end mode with a read length of 2x76bp. We generated minimally 65 million paired-end reads passing filter for each FFPE RNA-Seq library or at least 54 million paired-end reads passing filter for each FF RNA-Seq library in a fraction of a sequencing lane on HiSeq2000 (Illumina) following the manufacturer’s protocol. Image analysis, base calling and base quality scoring of the run were processed by integrated primary analysis software—Real Time Analysis (RTA 1.13.48) and followed by generation of FASTQ sequence files by CASAVA 1.8.

### Bioinformatics

The bioinformatic analyses included alignment and quantification, sample quality metrics, differential gene expression analysis, gene variant calling, and prediction of GBM molecular subtype.

#### Alignment and quantification

RNA-Seq reads were aligned to the human reference genome (GRCh38) using STAR (version 2.5.1b) [18] with ENCODE parameters for long RNA. The Y chromosome was removed from the reference genome to map the female samples. Genes were quantified using RSEM (version 1.2.28) [19] with default parameters. Human gene annotation file was downloaded from gencode release 24.

#### Sample quality metrics

Several quality metrics were calculated to evaluate the differences within each FF-FFPE pair and across the different preservation conditions. For categorical data,a Fisher’s exact test was applied for each pair. For differences in means between the two conditions, a t-test was applied. PCR duplicates were calculated with sambamba [20]. The number of detected genes was calculated taking into account genes with at least one paired-end read mapped. The number of genes consuming 25% of the reads was calculated by ranking the genes according to expression values (read counts) and then computing the cumulative sum until the number of reads was equal to 25% of the total sum. Mapping statistics were calculated with the tool ‘gtfcounts’ using GEMtools (http://gemtools.github.io/). Gene body coverage, GC content, paired-end inner distances, median transcript integrity number (TIN) across all the transcripts and distribution of mismatches across reads were computed with RSeQC [21, 22]. The percent spliced index (PSI) values were calculated with Spladder [23]. Correlation plots and principal component analysis were done with custom R scripts.

#### Differential gene sampling

RSEM read counts were used as input for DESeq2 (version 1.10.1) [24, 25]. The cut-off for considering a gene significantly up-sampled or down-sampled in the FFPE-derived samples was FDR<5%. Gene ontology enrichment analysis of the down-sampled FFPE genes was performed with DAVID database beta version 6.8 [26].

#### Gene variant calling

We counted the number of mismatches with respect to the reference genome for each GBM-associated gene directly from the mpileup generated by samtools without any filter [27, 28]. Variant calling was done with samtools with minimum base quality of 13, mapping quality >20, PCR duplicates removal, and minimum read depth of 10.

#### Prediction of GBM molecular subtype

The glmnet R package [29] was used to fit a multinomial logistic regression model with alpha = 1 lasso penalty. The cross-validation RNA-seq dataset was downloaded from the The Cancer Genome Atlas (TCGA) repository using the RTCGAToolbox R package (http://mksamur.github.io/RTCGAToolbox/). The core function ‘getFirehoseData’ with ‘dataset = GBM’ and ‘runDate = 20151101” was used to access and download the data. The associated clinical annotation for each sample was downloaded using the cgdsr R package (https://github.com/cBioPortal/cgdsr). The TCGA RNA-seq dataset comprised 145 RNA-seq samples grouped into the five established GBM molecular subtypes (Classical, Mesenchymal, Neural, Proneural and G-CIMP). Prediction was made for the four FF and the three informative FFPE samples (excluding FFPE_AA6365) using the largest value of lambda such that error was within 1 standard error of the minimum. Read counts were transformed with the variance stabilizing transformation using DESeq2.Batch effect correction between the RNA-seq datasets was carried out with the sva R package [30]. Genes with non-zero coefficient estimates were selected as best predictors.

### Data access

All data underlying the findings described in the manuscript are fully available without restriction from the BioProject database: https://www.ncbi.nlm.nih.gov/bioproject/342811.

## Results

### Quality and abundance of RNA

We had paired FF and FFPE samples from 11 patients. Of the 11 FF samples, only four met the requirements to ensure informative results from RNA-Seq (RIN≥6 and >50ng/μl or ≥1μg of total RNA). All analyses were carried out in the samples from these four patients. All FFPE RNAs had very low RIN values (≤2.6), but interestingly, RIN values were not related to storage time (Table 1).

**Table 1:** Characteristics of samples and sample selection.

	FF samples			FFPE samples		
Year	Pre-selection			Pre-selection		
	ng/μl	RIN	SAMPLE CODE	ng/μl	RIN	SAMPLE CODE
**2009**	7.09	1.1	UNSELECTED	163.47	2.5	UNSELECTED
**2008**	155.72	4.7		83.63	1.1	
**2011**	2.79	N/A		282.43	2.4	
**2009**	372.25	7.1	FF_AA6360	95.31	2.5	FFPE_AA6364
**2009**	489.17	8	FF_AA6361	321.86	2.4	FFPE_AA6365
**2007**	145.64	6.8	FF_AA6362	48.69	N/A	FFPE_AA6366
**2006**	549	7.3	FF_AA6363	1452.14	1.9	FFPE_AA6367
**2008**	66.3	2.4	UNSELECTED	115.3	1	UNSELECTED
**2008**	225.13	4.7		322.91	2.5	
**2009**	211.98	1.9		53.97	2.4	
**2010**	154.31	3.8		37.55	N/A	

All 11 paired samples were from patients with pathologically confirmed GBM. Gray shading indicates samples that were selected for analyses.

### Gradual degrees of degradation in FFPE samples

**Fig 1:**
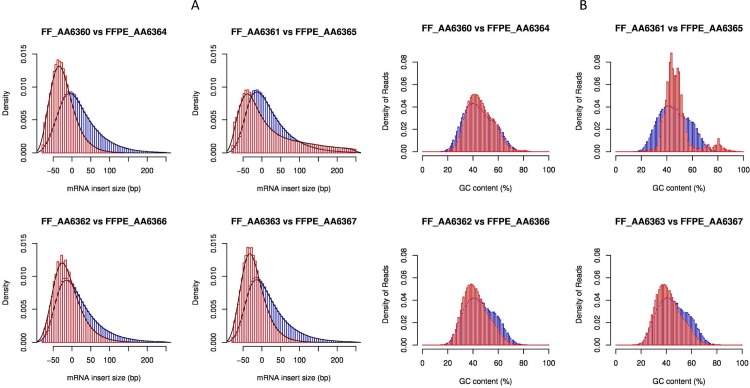
Degradation quality metrics in FF and FFPE tumour samples. (A) Paired-end distance distributions. Negative values correspond to overlapping paired-end reads. Blue lines represent FF samples and red lines represent FFPE samples. (B) Read GC content distributions. The more degraded the sample, the sharper the distribution. Regions with 40% of GC content are more conserved. A small peak at 80% of GC content can be clearly observed for the most degraded FFPE sample (AA6365). Blue lines represent FF samples and red lines represent FFPE samples.

**Fig 2:**
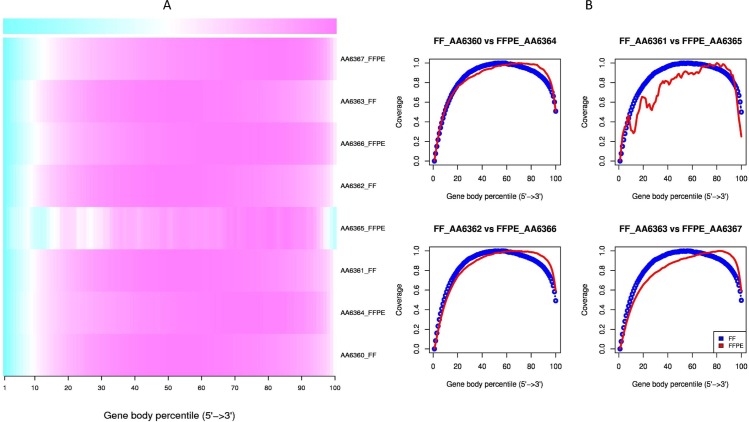
Degradation quality metrics. (A) Gene coverage heatmap. More degraded regions are depicted blue. All samples were affected at the 5’ end of the gene body but this effect was more prominent for FFPE samples. The most degraded FFPE sample (AA6365) also showed degradation at the 3’ end and across the gene body. (B) Line graphs (FF, blue; FFPE, red) showing the mean per-base coverage of RNA transcripts for all paired samples. Strong coverage unevenness was observed for the most degraded sample (FFPE_AA6365).

RNA fragmentation is a major effect of FFPE environments. A good proxy to evaluate if the RNA molecule length is affected is to calculate the paired-end inner distances for each RNA-seq experiment. As expected, FFPE specimens disclosed smaller distances between read pairs than FF samples (Fig 1A, P<0.001). In addition, the level of RNA degradation for each FFPE sample could be assessed by computing the transcript integrity number (TIN) [21]. Smaller TIN values were found for FFPE samples (P<0.001). Whereas FF samples had similar TIN values (mean TIN>60), the degree of degradation of FFPE RNA was very different among samples (Table 2). The most degraded FFPE sample (AA6365) had an extremely low value (mean TIN = 4), followed by a moderately degraded sample (AA63634, mean TIN = 29) and two less degraded samples (AA6366, mean TIN = 50; AA6367, mean TIN = 53). Degradation occurred more rapidly in regions with certain percentages of GC content (Fig 1B) and at the 5’ end of the transcripts (Fig 2A and 2B).

**Table 2:** Transcript integrity number (TIN) for paired FF and FFPE tumour samples.

		median	mean	standarddeviation
**pair 1**	**FF_AA6360**	72	63	27
	**FFPE_AA6364**	23	29	25
**pair 2**	**FF_AA6361**	72	64	27
	**FFPE_AA6365**	1	4	11
**pair 3**	**FF_AA6362**	73	64	27
	**FFPE_AA6366**	54	50	27
**pair 4**	**FF_AA6363**	72	64	27
	**FFPE_AA6367**	60	53	27

### Inferior library diversity in FFPE samples

**Fig 3:**
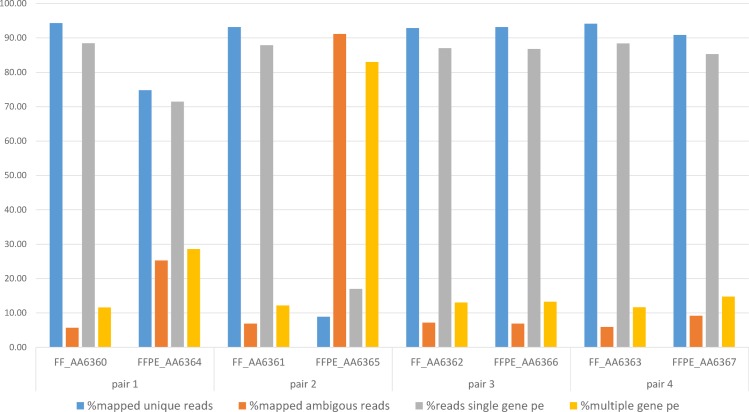
Mapped reads in FF and FFPE tissue samples. Percentages of uniquely mapped paired-reads, ambiguously mapped paired-end reads, paired-end reads mapping into a single gene, and paired-end reads mapping into multiple genes. Note that the most degraded FFPE sample (AA_6365) had very high percentages of ambiguous reads (>90%) and reads mapping to multiple genes (>80%), whereas the second most degraded FFPE sample (AA_6364) had intermediate percentages (25% and ~30% respectively). The remaining samples had low percentages of ambiguities (~10%).

Investigating the library diversity captured by sequencing FFPE material can help identify any loss of informative RNA-Seq reads due to poor sampling of the RNA molecules [31, 32]. We first examined library diversity based on the duplication rate. As expected and as described elsewhere [6, 10], FFPE samples presented higher percentages of duplicates than their matched FF samples (all pairs P<2.2x10^-16^, Table 3, S1 File). Consistent with these findings, there was a greater decrease in the number of uniquely mapped reads in the more degraded samples (all pairs P<2.2^2x10-16^ except pair 3, Fig 3, S1 File). We also examined library diversity by determining the number of genes needed to consume 25% of the sequencing effort. In general, fewer genes were needed for FFPE samples than for their matched FF samples (all pairs P<2.2x10^-16^ except pair 3, Table 3, S1 File). Interestingly, this number was extremely low for the most degraded FFPE sample (AA_6365), where onegene accounted for 25% of the sequencing effort. Not surprisingly, the number of genes in this sample was much lower (~8000 genes) than in the other samples (~25,000–30,000), and it also harboured the highest percentage (>90%) of ambiguously mapped reads (Fig 3). These results suggest that the most highly degraded FFPE libraries are enriched with a few extremely dominant genes and are therefore less diverse.

**Table 3:** Library diversity quality metrics.

		percentage of duplicates	number of genes detected	number of genes consuming 25% of sequencing
**pair 1**	**FF_AA6360**	12.62	28763	109
	**FFPE_AA6364**	45.41	25900	11
**pair 2**	**FF_AA6361**	19.37	27511	64
	**FFPE_AA6365**	29.36	8239	1
**pair 3**	**FF_AA6362**	13.83	29771	56
	**FFPE_AA6366**	27.29	28676	66
**pair 4**	**FF_AA6363**	15.36	28394	98
	**FFPE_AA6367**	20.85	28518	75

### RNA molecules are better preserved in the mitochondria and nucleus than in the cytosol of FFPE samples

**Fig 4:**
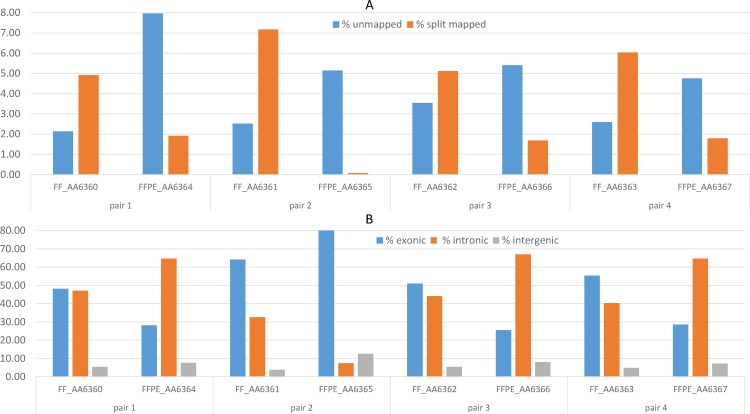
Mapping results in FFPE and matched FF tissue samples. (A) Percentages of unmapped reads and split-mapped reads in FFPE and FF samples. (B) Percentages of paired-end reads mapping to exonic, intronic or intergenic regions.

**Fig 5:**
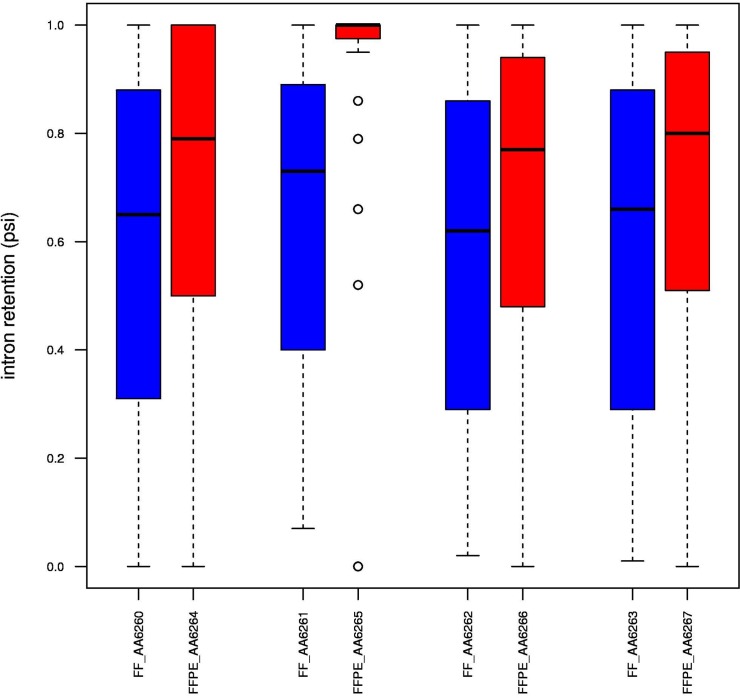
Boxplots of PSI values for intron retention events. Results for FF samples are shown in blue and those for FFPE samples in red. The PSI value was defined as the number of reads supporting the inclusion divided by the number of reads supporting the inclusion or the exclusion. The median PSI value for intron retention events was higher in FFPE samples, suggesting a greater abundance of transcripts with unspliced introns, such as pre-mRNAs or linc-RNAs.

As previously reported [15], mapping quality metrics showed slightly higher percentages of unmapped reads and lower percentages of splice-mapped reads in FFPE samples than in the matched FF samples (all pairs P<2.2x10^-16^, Fig 4A, S1 File). All FFPE and FF samples showed a higher number of reads mapping to introns than to exons, a common result with RiboZero RNA-Seq protocols [10], but this effect was even more pronounced in FFPE samples (all pairs P<2.2x10^-16^, Fig 4B, S1 File). We speculated that this might be due to the fact that spliced transcripts in the cytosol are more susceptible to degradation, while intron-rich features, such as pre-mRNA or lincRNA, in the nucleus remain protected. To test this hypothesis, we calculated the percent spliced index (PSI) for each sample and observed a higher median value for FFPE samples (Fig 5), confirming that we were dealing with higher fractions of pre-mRNA with unspliced introns.

**Fig 6:**
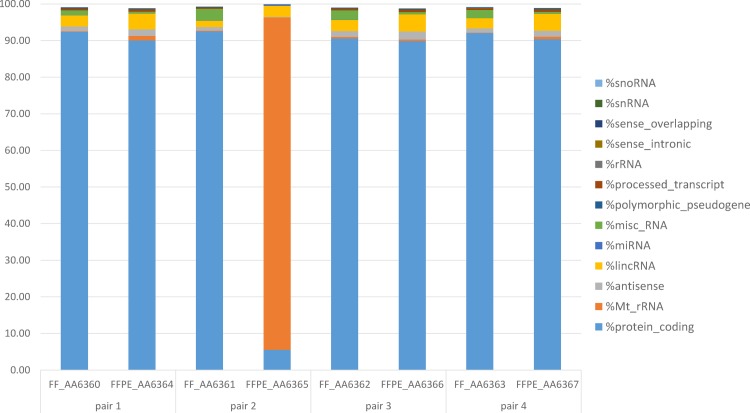
Annotated paired-end reads mapping to different gene biotypes. The majority of annotated reads mapped to protein-coding genes for all samples except FFPE_AA6365, which showed extremely high amounts of ribosomal MT RNA. The percentage of reads mapping to non-coding RNA was higher for FFPE than FF samples.

Fig 6 displays the annotated paired-end reads mapping to different gene biotypes in the matched FF and FFPE tumor samples. In both FF and FFPE samples, the majority of the annotated paired-end reads mapped to the protein-coding gene biotype (~90%), though with a slightly higher percentage in FF samples (all pairs P<2.2x10^-16^, S1 File). In contrast, the non-coding RNA biotypes, such as lincRNA and snRNA, showed higher percentages of reads in FFPE than FF samples. Interestingly, however, in the most degraded FFPE sample (AA_6365) only 5% of reads mapped to protein-coding genes, while 90% mapped to mitochondrial rRNA, which may be due to a better preservation of mitochondria organelles in the context of a degradation-prone FFPE environment. (Related statistical analyses are shown in S1 File.)

Differential gene expression analysis revealed 2133 differentially sampled genes with FDR<0.05 (S2 File). In FFPE samples, 908 protein-coding genes and 26 non-coding RNAs were down-sampled, whereas 169 protein-coding genes and 1030 non-coding RNAs were over-sampled (Table 4). Over-sampled FFPE genes were either non-coding genes transcribed in the nucleus and not transported in the cytosol, such as RNU, SCARNA, SNORA, and LINC families, or those transcribed in the mitochondria, such as MT-ATP, MT-ND, MT-CO families and many MT pseudogenes. Importantly, the majority of these protected genes have no functional annotation. In contrast, down-sampled FFPE genes were nuclear-encoded and actively translated mRNA in the cytosol. The biological processes enriched in down-sampled FFPE RNAs included translation (RPL and RPS ribosomal genes), generation of precursor metabolites and energy (nuclear-encoded MT genes), DNA packaging (HIST genes), RNA processing (POLR and SNRP genes), proteosomal catabolic process (PSM genes), cell cycle (TUBB) and protein folding (HSP and CTT genes) (S2 File).

**Table 4:** Number of over-sampled and down-sampled gene biotypes in FFPE specimens.

Gene biotype	Over-sampled	Down-sampled
protein-coding	169	908
sense-intronic	167	1
lincRNA	161	3
processed pseudogene	137	9
TEC	133	0
antisense	115	2
miscellaneous_RNA	89	0
snoRNA	56	1
snRNA	44	0
processed transcript	30	1
unprocessed_pseudogene	24	2
transcribed_unprocessed_pseudogene	23	2
sense-overlapping	18	0
transcribed_processed_pseudogene	15	3
miRNA	6	0
scaRNA	6	0
mitochondrial_rRNA	2	0
unitary_pseudogene	2	0
non-coding	1	0
rRNA	1	0
ribozyme	0	2

### High similarities in gene expression between FF and less degraded FFPE samples

**Fig 7:**
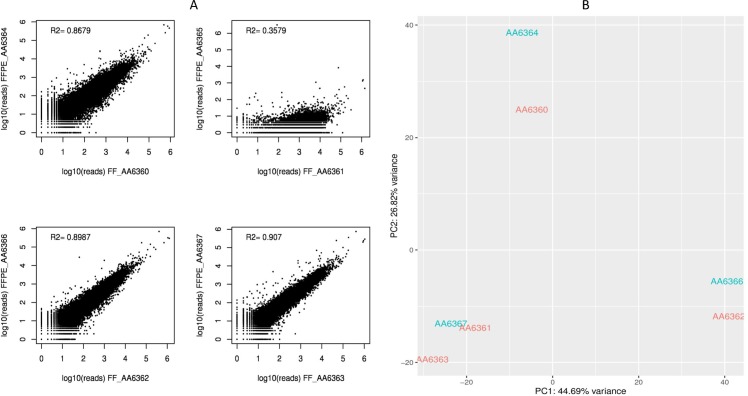
Comparison of gene expression between FF and FFPE samples. (A) Correlation plots of gene expression in FF-FFPE pairs. In general, the correlation was high (R^2^~0.9), with the exception of the FF_AA6361-FFPE_AA6365 pair, where the FFPE sample was highly degraded. Higher variability was observed for more degraded samples. (B) Results of the principal component analysis. FF-FFPE pairs clustered together. The most degraded sample (FFPE_AA6365) was not included in the plot.

In spite of several differences in the quality metrics between FF and FFPE samples, the correlation of gene expression within each pair was high (R^2^~0.9), with the exception of FFPE_AA6365, the most highly degraded sample (R^2^~0.35) (Fig 7A). A principal component analysis showed that paired samples clustered closely together, thus indicating conserved similarities in gene expression (Fig 7B).

### Distinct mismatch profiles in FFPE and FF samples

**Fig 8:**
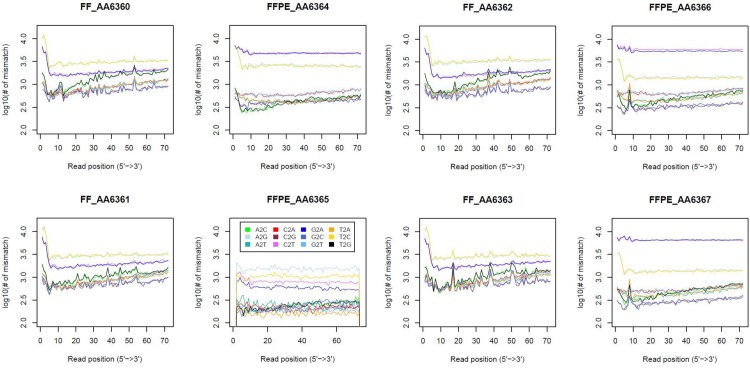
Number of mismatches across the read length. Mismatch profiles changed dramatically mainly due to G>A and C>T changes, which were substantially more frequent in FFPE samples (top pink and blue lines). Sample FFPE_AA6365, which was highly degraded, showed a totally different pattern, not matching with any other sample.

The FFPE mismatch profiles diverged substantially from their paired FF samples (Fig 8). Specifically, G>A and C>T transitions were much more frequent in FFPE samples. These two nucleotide changes have already been reported in other FFPE studies [15] and have been described as a chemical artefact caused during the paraffin fixation process. The six commonly mutated GBM genes (*IDH1*, *IDH2*, *NF1*, *PTEN*, *PDGFRA* and *TP53*) [1, 3, 33] also harboured many of these mutational artefacts (S1 Table). Although Graw et al [15] reported that these paraffin mutations appear at low frequencies, affecting few RNA molecules, in some cases we only found the mutated allele. Single nucleotide polymorphisms (SNPs) called in GBM-associated genes with G>A and C>T transitions are shown in S2 Table. In addition to FFPE chemical artefacts, differences in SNPs between the paired samples (S3 Table) may be due to differential read depth. For example, the gene may be partially degraded in FFPE, making it impossible to recover the SNP.

### Heterogeneity in GBM molecular subtypes

The prediction of molecular subtype with Lasso regularization showed that all but one FFPE sample (AA6365) could be classified in one of the five GBM molecular subtypes [3] (Table 5). However, the predictive ability of the model was quite low (mean cross-validated error 33.7% +/- SD 3.9%), which might be due to the high degree of heterogeneity of GBM tumors [34]. The mesenchymal subtype was assigned with a slightly higher level of confidence (prob = 0.40–0.49) than the proneural (prob = 0.28–0.36) and classical subtypes (prob = 0.33). As the GBM samples were extracted from different locations within the tumour, not unexpectectly, there was one discrepancy in one FF-FFPE pair. None of the samples were assigned to the neural or G-CIMP subtypes. From the 38 predictors selected by the model, ten overlapped with the Verhaak 840-gene signature [1] (S4 Table).

**Table 5:** Prediction of GBM molecular subtypes.

		Predicted	Classical	G-CIMP	Mesenchymal	Neural	Proneural
**pair 1**	FF_AA6360	Proneural	0.23	0.09	0.27	0.08	**0.32**
	FFPE_AA6364	Mesenchymal	0.20	0.06	**0.43**	0.11	0.21
**pair 2**	FF_AA6361	Classical	**0.33**	0.04	0.31	0.11	0.21
	FFPE_AA6365	NA	NA	NA	NA	NA	NA
**pair 3**	FF_AA6362	Proneural	0.16	0.13	0.18	0.16	**0.36**
	FFPE_AA6366	Proneural	0.20	0.10	0.23	0.18	**0.28**
**pair 4**	FF_AA6363	Mesenchymal	0.25	0.02	**0.49**	0.09	0.15
	FFPE_AA6367	Mesenchymal	0.22	0.05	**0.40**	0.15	0.17

Numbers represent probabilities. The predicted GBM molecular subtype is based on fitted class probabilities. The highest class probability is depicted in bold.

## Discussion

GBM is a rare disease (http://www.rarecancerseurope.org//About-Rare-Cancer, https://www.ncbi.nlm.nih.gov/pmc/articles/PMC2789814/) with an incidence in adults of 3.19 per 100,000 inhabitants and a high mortality rate[35]. Genomic investigation is crucial to improving patient outcome, but there are a number of obstacles to overcome in the investigation of GBM. First of all, in GBM, as in all rare cancers, it is difficult to obtain an appropriate number of samples with sufficient follow-up to enable investigators to draw reliable conclusions on prognosis and treatment outcomes. Multi-institutional collaboration can increase the number of subjects with available samples and is the key to obtaining dependable statistical results. A further problem in glioblastoma is that tissue obtained from surgery is scarce and histologic diagnosis is prioritized to fresh tissue storage, which reduces the number of FF specimens available for investigation. In addition, only 50% of patients receive standard treatment–often due to low performance status or older age–which further reduces the possibility of obtaining comprehensive data on disease progression and patient outcome [36]. In this setting, FFPE tissues can provide a large volume of biospecimens and may thus represent an opportunity to investigate genetic changes that drive clinical outcome. However, it is not clear whether genomic data obtained from FFPE tissue is as reliable as that obtained from FF tissue. In the present study, we have found that although many FFPE samples were highly degraded and thus could not be included in the study, RNA from those FFPE samples that were not degraded maintained transcriptomic similarities to that obtained from FF samples.

The GLIOCAT project recruited patients with GBM who had all been treated with the standard treatment of radiotherapy with concomitant and adjuvant temozolomide, who had clinical information available, and for whom there was sufficient FFPE tumour tissue to perform genomic studies. Of 432 patients included in the GLIOCAT project, 247 had sufficient RNA extracted from FFPE samples to proceed with Illumina RNA-Seq. Nevertheless, before launching the RNA-Seq analyses in the entire cohort, we performed the present pilot feasibility study to determine if results obtained by RNA-Seq of FFPE samples would be completely reliable. We therefore selected those patients for whom we had both FF and FFPE samples from the same tumour. Only 11 patients met these criteria, all of whom were from six university hospitals, each of which had its own biobank.

Other studies have examined the correlation between FF and FFPE samples. Graw et al [15] compared matched FF and FFPE ovarian tumour samples with Illumina RNA-Seq. In line with our results, they also found the FFPE mutational artefacts G>A and C>T, but at low allele frequencies (AF<0.5) and they applied an AF filter to remove them. In contrast, we found some of these artefacts at very high frequencies (AF = 0.5–1). Moreover, the artefacts observed in our study affected GBM-associated genes, which would pose a problem for detecting somatic mutations in the FFPE samples. In addition, the differences reported on gene coverage, GC content, read mapping, and quality metrics could be due to the different protocols they used to analyze the samples (mRNA-Seq for FF samples and RiboZero total RNA for FFPE samples) [15]. Nevertheless, in the present study, the differences can be attributable only to inherent differences in FFPE compared to FF, as the protocol used for RNA-Seq was the same in both types of samples. Gravendeel et al [37] performed expression profiling on 55 paired FF-FFPE glioma samples using HUI 33 plus 2.0 arrays in FF samples and Human Exon 1.0 ST arrays in FFPE samples. Although in general, the correlation between FF and FFPE expression was poor, when they selected the most variable probe sets on FFPE expression profiles, concordance improved. Moreover, with the selected probe sets, they were able to correctly assign 87% of the FFPE samples to one of the seven glioma subtypes they had previously identified using FF samples [2]. They attribute variability in their findings to tumour heterogeneity REF. In a third study, Erdem-Eraslan et al [17] performed RNA-Seq in FF and matched FFPE GBM samples and were able to correctly assign 100% of their 114 samples to a molecular subgroup–either Gravendeel’s [2] or Verhaaak’s [1]–using the ClusterRepro R package. Previously, they had run a series of tests to determine the suitability of DASL arrays and RNA-Seq on RNA isolated from FFPE tissues, comparing technical and biological replicates with those obtained from paired FF samples. They found that both FFPE and FF tissues could be used to perform gene expression profiling, although they did not provide details on how many samples were uninformative or on whether the two types of tissue provided similar information [17]. To the best of our knowledge, our study provides the first in-depth comparison of information obtained with RNA-Seq in paired FF and FFPE GBM samples. In our experience, the RNA isolated from FFPE samples was highly degraded. In fact, RNA quantity and quality was low even in FF samples, as only four of eleven samples met the requirements to ensure informative results with RNA-Seq. We can conclude that even in FF GBM samples, RNA can only be extracted in low amounts with low integrity levels, which further impedes genomic sequencing in GBM.

In our study, we found high variability in the degree of RNA degradation in FFPE samples. Nevertheless, once the more degraded samples were excluded by transcriptomic quality control, FFPE samples showed transcriptomic similarities and high correlation of gene expression with FF samples. Differences in gene expression did not preclude the classification of the specimens into established GBM molecular subtypes, albeit at a low confidence level. In fact, tumour heterogeneity is a major issue for molecular classification [34, 38]. The study of somatic mutations remains a challenge in both FF and FFPE tissues, as healthy tissue is needed to identify them in FF samples and, conversely, it is difficult to identify them beyond a doubt in FFPE samples due to the presence of artefacts. Nevertheless, the RNA molecules inside the nucleus and the mitochondria seem to be protected in FFPE tissues, indicating that FFPE samples can be useful for investigating the non-coding part of the genome.

## Conclusion

Our results suggest that archival FFPE material can be used for RNA-Seq analysis of GBM specimens if the RNA is sufficiently preserved, but the majority of samples are too degraded to provide fully informative results. This issue underscores the need for multi-institutional collaboration in order to gather a sufficient number of samples, especially in rare diseases like GBM, to draw reliable conclusions from genomic analyses. Moreover, in an era of genomic-based studies, efforts are warranted to improve methods of tissue storage in order to preserve genomic information.

## Supporting Information

S1 File Statistical results. Results of statistical analyses of quality metrics. A one-tailed Fisher’s exact test was applied to each FF and FFPE pair.
(XLSX)

S2 File Excel sheets. There are five tabs: (A) Results of the differential gene sampling analysis. Genes are sorted by significance. (B) List of differentially sampled genes with FDR <0.05. (C) List of over-sampled genes in FFPE samples sorted by gene name. The majority are non-coding RNA belonging to specific gene families and have no functional annotation. (D) List of down-sampled genes in FFPE sorted by gene name. The majority are protein-coding genes. (E) Gene ontology enrichment
(XLSX)

S1 Table mpileup changes in GBM associated genes. Number of mpileup substitutions with respect to the reference genome. Gray shaded areas indicate C>T and G>A changes.
(DOCX)

S2 Table C>T and G>A FFPE artefacts in GBM-associated genes. Number of SNP artefacts originated by C>T and G>A changes. We counted cases that were CC (GG) homozygous in the FF sample and CT (GA) or TT (AA) in the paired FFPE sample.
(DOCX)

S3 Table Overlapping and non-overlapping SNPs in FF-FFPE pairs. Number of overlapping SNPs (shaded) and non-overlapping SNPs (non-shaded) for each FF-FFPE pair and each gene. 0/1 indicates a heterozygous SNP. 1/1 indicates a homozygous alternative. NA indicates not assessed.
(DOCX)

S4 Table Best gene predictors of GBM molecular classification. Grey shaded areas indicate genes included in Verhaak’s gene signature.
(DOCX)
