# Supplementary material for: A Comparison of RNA-Seq Results from Paired Formalin-Fixed Paraffin-Embedded and Fresh-Frozen Glioblastoma Tissue Samples
Source: PLoS One. 2017 Jan 25;12(1):e0170632. doi: 10.1371/journal.pone.0170632 (PMC5266269; doi:10.1371/journal.pone.0170632)
Supplement: S1 Table — Number of mpileup substitutions with respect to the reference genome. Gray shaded areas indicate C>T and G>A changes. (DOCX) [file pone.0170632.s003.docx]

**S1 Table. mpileup changes in GBM associated genes.** Number of mpileup substitutions with respect to the reference genome. Gray shaded areas indicate C>T and G>A changes.

|  | **Fresh-frozen samples** | | | | **Formalin-fixed paraffin-embedded samples** | | | |
| --- | --- | --- | --- | --- | --- | --- | --- | --- |
|  | FF_AA6360 | FF_AA6361 | FF_AA6362 | FF_AA6363 | FFPE_AA6364 | FFPE_AA6365 | FFPE_AA6366 | FFPE_AA6367 |
| ***TP53*** | | | | | | | | |
| A>C | 6 | 2 | 7 | 12 | 5 | NA | 9 | 39 |
| A>G | 18 | 5 | 25 | 42 | 17 | NA | 10 | 77 |
| A>T | 3 | 1 | 7 | 12 | 4 | NA | 18 | 44 |
| C>A | 9 | 2 | 7 | 12 | 6 | NA | 32 | 48 |
| C>G | 8 | 2 | 6 | 9 | 5 | NA | 10 | 33 |
| **C>T** | **14** | **3** | **22** | **31** | **27** | NA | **20** | **83** |
| **G>A** | **12** | **7** | **13** | **33** | **95** | NA | **185** | **859** |
| G>C | 6 | 2 | 4 | 14 | 6 | NA | 6 | 20 |
| G>T | 9 | 0 | 6 | 10 | 9 | NA | 25 | 55 |
| T>A | 6 | 2 | 2 | 7 | 2 | NA | 16 | 45 |
| T>C | 37 | 15 | 29 | 63 | 43 | NA | 34 | 139 |
| T>G | 5 | 1 | 2 | 8 | 2 | NA | 11 | 44 |
| ***PTEN*** | | | | | | | | |
| A>C | 13 | 13 | 10 | 13 | 16 | NA | 39 | 104 |
| A>G | 100 | 88 | 88 | 72 | 96 | NA | 97 | 207 |
| A>T | 26 | 31 | 24 | 22 | 40 | NA | 76 | 126 |
| C>A | 17 | 12 | 13 | 15 | 29 | NA | 45 | 72 |
| C>G | 9 | 8 | 8 | 6 | 9 | NA | 10 | 32 |
| **C>T** | **37** | **38** | **25** | **41** | **638** | NA | **1086** | **2672** |
| **G>A** | **50** | **36** | **35** | **50** | **54** | NA | **49** | **98** |
| G>C | 20 | 25 | 8 | 16 | 20 | NA | 29 | 46 |
| G>T | 23 | 23 | 12 | 25 | 54 | NA | 80 | 158 |
| T>A | 32 | 28 | 18 | 19 | 21 | NA | 50 | 111 |
| T>C | 120 | 103 | 80 | 81 | 79 | NA | 71 | 154 |
| T>G | 35 | 14 | 14 | 18 | 37 | NA | 57 | 83 |
| ***PDGFRA*** | | | | | | | | |
| A>C | 12 | 17 | 11 | 7 | 0 | NA | 9 | 23 |
| A>G | 56 | 118 | 37 | 30 | 15 | NA | 19 | 50 |
| A>T | 19 | 48 | 15 | 10 | 6 | NA | 11 | 27 |
| C>A | 15 | 27 | 13 | 11 | 4 | NA | 12 | 21 |
| C>G | 10 | 28 | 8 | 15 | 3 | NA | 5 | 9 |
| **C>T** | **28** | **99** | **26** | **20** | **84** | NA | **294** | **739** |
| **G>A** | **42** | **88** | **30** | **39** | **6** | NA | **20** | **39** |
| G>C | 11 | 32 | 15 | 10 | 7 | NA | 8 | 18 |
| G>T | 16 | 33 | 8 | 9 | 6 | NA | 20 | 41 |
| T>A | 18 | 43 | 12 | 7 | 5 | NA | 8 | 17 |
| T>C | 67 | 151 | 56 | 45 | 8 | NA | 21 | 44 |
| T>G | 14 | 36 | 16 | 8 | 2 | NA | 18 | 18 |
| ***NF1*** | | | | | | | | |
| A>C | 64 | 27 | 43 | 41 | 41 | NA | 225 | 281 |
| A>G | 457 | 329 | 598 | 392 | 456 | NA | 725 | 1029 |
| A>T | 72 | 41 | 87 | 78 | 52 | NA | 325 | 451 |
| C>A | 60 | 41 | 63 | 47 | 97 | NA | 268 | 283 |
| C>G | 53 | 33 | 46 | 37 | 38 | NA | 113 | 108 |
| **C>T** | **135** | **103** | **117** | **109** | **908** | NA | **5219** | **7665** |
| **G>A** | **192** | **139** | **148** | **159** | **209** | NA | **461** | **617** |
| G>C | 77 | 43 | 60 | 53 | 51 | NA | 178 | 191 |
| G>T | 70 | 48 | 58 | 56 | 70 | NA | 371 | 402 |
| T>A | 82 | 53 | 80 | 69 | 112 | NA | 285 | 318 |
| T>C | 334 | 219 | 272 | 263 | 156 | NA | 403 | 497 |
| T>G | 85 | 47 | 58 | 62 | 74 | NA | 250 | 295 |
| ***IDH1*** | | | | | | | | |
| A>C | 18 | 7 | 3 | 11 | 2 | NA | 16 | 30 |
| A>G | 55 | 28 | 19 | 29 | 11 | NA | 21 | 45 |
| A>T | 13 | 6 | 7 | 10 | 6 | NA | 20 | 14 |
| C>A | 15 | 7 | 4 | 3 | 4 | NA | 21 | 27 |
| C>G | 13 | 1 | 3 | 2 | 4 | NA | 5 | 13 |
| **C>T** | **28** | **17** | **17** | **15** | **9** | NA | **20** | **21** |
| **G>A** | **25** | **21** | **14** | **10** | **84** | NA | **270** | **529** |
| G>C | 13 | 3 | 2 | 7 | 1 | NA | 7 | 6 |
| G>T | 10 | 8 | 3 | 8 | 9 | NA | 29 | 18 |
| T>A | 20 | 13 | 2 | 3 | 5 | NA | 22 | 36 |
| T>C | 28 | 18 | 15 | 20 | 16 | NA | 29 | 32 |
| T>G | 14 | 5 | 3 | 8 | 1 | NA | 8 | 19 |
| ***IDH2*** | | | | | | | | |
| A>C | 16 | 11 | 14 | 19 | 10 | NA | 41 | 34 |
| A>G | 55 | 22 | 52 | 50 | 12 | NA | 51 | 35 |
| A>T | 9 | 5 | 8 | 8 | 5 | NA | 31 | 21 |
| C>A | 14 | 4 | 17 | 17 | 9 | NA | 63 | 40 |
| C>G | 19 | 10 | 21 | 11 | 9 | NA | 23 | 24 |
| **C>T** | **38** | **23** | **63** | **44** | **23** | NA | **55** | **57** |
| **G>A** | **21** | **21** | **43** | **33** | **90** | NA | **483** | **659** |
| G>C | 8 | 6 | 16 | 14 | 3 | NA | 14 | 20 |
| G>T | 14 | 9 | 20 | 15 | 12 | NA | 49 | 38 |
| T>A | 10 | 3 | 10 | 12 | 2 | NA | 35 | 28 |
| T>C | 54 | 12 | 86 | 67 | 31 | NA | 58 | 68 |
| T>G | 10 | 5 | 8 | 11 | 5 | NA | 30 | 40 |
