# Supplementary material for: A Comparison of RNA-Seq Results from Paired Formalin-Fixed Paraffin-Embedded and Fresh-Frozen Glioblastoma Tissue Samples
Source: PLoS One. 2017 Jan 25;12(1):e0170632. doi: 10.1371/journal.pone.0170632 (PMC5266269; doi:10.1371/journal.pone.0170632)
Supplement: S2 Table — Number of SNP artefacts originated by C>T and G>A changes. We counted cases that were CC (GG) homozygous in the FF sample and CT (GA) or TT (AA) in the paired FFPE sample. (DOCX) [file pone.0170632.s004.docx]

**S2 Table. C>T and G>A FFPE artefacts in GBM-associated genes.** Number of SNP artefacts originated by C>T and G>A changes. We counted cases that were CC (GG) homozygous in the FF sample and CT (GA) or TT (AA) in the paired FFPE sample.

|  | FF_AA6360-FFPE_AA6364 | FF_AA6361-FFPE_AA6365 | FF_AA6362-FFPE_AA6366 | FF_AA6363-FFPE_AA6367 |
| --- | --- | --- | --- | --- |
| ***TP53*** | | | | |
| C>T | 0 | NA | 0 | 0 |
| G>A | 0 | NA | 0 | 0 |
| ***PTEN*** | | | | |
|  | FF_AA6360-FFPE_AA6364 | FF_AA6361-FFPE_AA6365 | FF_AA6362-FFPE_AA6366 | FF_AA6363-FFPE_AA6367 |
| C>T | 0 | NA | 0 | 0 |
| G>A | 2 | NA | 1 | 1 |
| ***PDGFRA*** | | | | |
|  | FF_AA6360-FFPE_AA6364 | FF_AA6361-FFPE_AA6365 | FF_AA6362-FFPE_AA6366 | FF_AA6363-FFPE_AA6367 |
| C>T | 0 | NA | 0 | 1 |
| G>A | 0 | NA | 0 | 0 |
| ***NF1*** | | | | |
|  | FF_AA6360-FFPE_AA6364 | FF_AA6361-FFPE_AA6365 | FF_AA6362-FFPE_AA6366 | FF_AA6363-FFPE_AA6367 |
| C>T | 0 | NA | 8 | 9 |
| G>A | 1 | NA | 6 | 14 |
| ***IDH1*** | | | | |
|  | FF_AA6360-FFPE_AA6364 | FF_AA6361-FFPE_AA6365 | FF_AA6362-FFPE_AA6366 | FF_AA6363-FFPE_AA6367 |
| C>T | 0 | NA | 0 | 0 |
| G>A | 0 | NA | 0 | 0 |
| ***IDH2*** | | | | |
|  | FF_AA6360-FFPE_AA6364 | FF_AA6361-FFPE_AA6365 | FF_AA6362-FFPE_AA6366 | FF_AA6363-FFPE_AA6367 |
| C>T | 0 | NA | 1 | 0 |
| G>A | 0 | NA | 1 | 2 |
