# Supplementary material for: A Comparison of RNA-Seq Results from Paired Formalin-Fixed Paraffin-Embedded and Fresh-Frozen Glioblastoma Tissue Samples
Source: PLoS One. 2017 Jan 25;12(1):e0170632. doi: 10.1371/journal.pone.0170632 (PMC5266269; doi:10.1371/journal.pone.0170632)
Supplement: S3 Table — Number of overlapping SNPs (shaded) and non-overlapping SNPs (non-shaded) for each FF-FFPE pair and each gene. 0/1 indicates a heterozygous SNP. 1/1 indicates a homozygous alternative. NA indicates not assessed. (DOCX) [file pone.0170632.s005.docx]

**S3 Table. Overlapping and non-overlapping SNPs in FF-FFPE pairs.** Number of overlapping SNPs (shaded) and non-overlapping SNPs (non-shaded) for each FF-FFPE pair and each gene. 0/1 indicates a heterozygous SNP. 1/1 indicates a homozygous alternative. NA indicates not assessed.

|  | **FF_AA6360-FFPE_AA6364** | **FF_AA6360-FFPE_AA6365** | **FF_AA6362-FFPE_AA6366** | **FF_AA6363-FFPE_AA6367** |
| --- | --- | --- | --- | --- |
| ***TP53*** | | | | |
| 0/1-0/1 | 0 | NA | 0 | 0 |
| 0/1-1/1 | 0 | NA | 0 | 0 |
| 0/1-NA | 0 | NA | 0 | 0 |
| 1/1-1/1 | 0 | NA | 0 | 1 |
| 1/1-0/1 | 0 | NA | 0 | 0 |
| 1/1-NA | 0 | NA | 0 | 0 |
| NA-0/1 | 0 | NA | 0 | 6 |
| NA-1/1 | 0 | NA | 0 | 1 |
| ***PTEN*** | | | | |
| 0/1-0/1 | 0 | NA | 1 | 0 |
| 0/1-1/1 | 0 | NA | 0 | 1 |
| 0/1-NA | 6 | NA | 1 | 0 |
| 1/1-1/1 | 0 | NA | 0 | 0 |
| 1/1-0/1 | 0 | NA | 0 | 1 |
| 1/1-NA | 4 | NA | 1 | 0 |
| NA-0/1 | 4 | NA | 1 | 3 |
| NA-1/1 | 1 | NA | 0 | 0 |
| ***PDGFRA*** | | | | |
| 0/1-0/1 | 0 | NA | 0 | 2 |
| 0/1-1/1 | 0 | NA | 0 | 0 |
| 0/1-NA | 0 | NA | 0 | 1 |
| 1/1-1/1 | 2 | NA | 4 | 4 |
| 1/1-0/1 | 0 | NA | 0 | 0 |
| 1/1-NA | 3 | NA | 1 | 0 |
| NA-0/1 | 0 | NA | 0 | 1 |
| NA-1/1 | 0 | NA | 0 | 0 |
| ***NF1*** | | | | |
| 0/1-0/1 | 2 | NA | 23 | 28 |
| 0/1-1/1 | 0 | NA | 0 | 0 |
| 0/1-NA | 66 | NA | 21 | 10 |
| 1/1-1/1 | 2 | NA | 1 | 0 |
| 1/1-0/1 | 1 | NA | 0 | 0 |
| 1/1-NA | 9 | NA | 1 | 0 |
| NA-0/1 | 15 | NA | 51 | 75 |
| NA-1/1 | 1 | NA | 0 | 7 |
| ***IDH1*** | | | | |
| 0/1-0/1 | 0 | NA | 0 | 0 |
| 0/1-1/1 | 0 | NA | 0 | 0 |
| 0/1-NA | 0 | NA | 0 | 0 |
| 1/1-1/1 | 1 | NA | 3 | 1 |
| 1/1-0/1 | 0 | NA | 0 | 0 |
| 1/1-NA | 8 | NA | 1 | 3 |
| NA-0/1 | 0 | NA | 0 | 0 |
| NA-1/1 | 0 | NA | 0 | 2 |
| ***IDH2*** | | | | |
| 0/1-0/1 | 0 | NA | 2 | 0 |
| 0/1-1/1 | 0 | NA | 0 | 0 |
| 0/1-NA | 4 | NA | 3 | 0 |
| 1/1-1/1 | 1 | NA | 0 | 2 |
| 1/1-0/1 | 0 | NA | 0 | 0 |
| 1/1-NA | 1 | NA | 0 | 0 |
| NA-0/1 | 0 | NA | 6 | 5 |
| NA-1/1 | 0 | NA | 2 | 1 |

NA: not assessed.
