# Supplementary material for: A Comparison of RNA-Seq Results from Paired Formalin-Fixed Paraffin-Embedded and Fresh-Frozen Glioblastoma Tissue Samples
Source: PLoS One. 2017 Jan 25;12(1):e0170632. doi: 10.1371/journal.pone.0170632 (PMC5266269; doi:10.1371/journal.pone.0170632)
Supplement: S4 Table — Grey shaded areas indicate genes included in Verhaak’s gene signature. (DOCX) [file pone.0170632.s006.docx]

**S4 Table. Best gene predictors of GBM molecular classification.** Grey shaded areas indicate genes included in Verhaak’s gene signature.

| Ensembl Gene ID | Associated Gene Name |
| --- | --- |
| ENSG00000011028 | **MRC2*** |
| ENSG00000039560 | RAI14 |
| ENSG00000064012 | **CASP8*** |
| ENSG00000074410 | CA12 |
| ENSG00000079101 | CLUL1 |
| ENSG00000099250 | **NRP1*** |
| ENSG00000103642 | LACTB |
| ENSG00000105072 | C19orf44 |
| ENSG00000108591 | DRG2 |
| ENSG00000110429 | **FBXO3*** |
| ENSG00000114115 | RBP1 |
| ENSG00000115350 | POLE4 |
| ENSG00000118971 | CCND2 |
| ENSG00000119283 | TRIM67 |
| ENSG00000119681 | **LTBP2*** |
| ENSG00000121417 | **ZNF211*** |
| ENSG00000127955 | **GNAI1*** |
| ENSG00000133794 | **ARNTL*** |
| ENSG00000135074 | **ADAM19*** |
| ENSG00000137460 | FHDC1 |
| ENSG00000142449 | FBN3 |
| ENSG00000142700 | DMRTA2 |
| ENSG00000145779 | **TNFAIP8*** |
| ENSG00000151005 | TKTL2 |
| ENSG00000154124 | OTULIN |
| ENSG00000155962 | CLIC2 |
| ENSG00000158887 | MPZ |
| ENSG00000160179 | ABCG1 |
| ENSG00000162949 | CAPN13 |
| ENSG00000169045 | HNRNPH1 |
| ENSG00000170144 | HNRNPA3 |
| ENSG00000173559 | NABP1 |
| ENSG00000176294 | OR4N2 |
| ENSG00000177108 | ZDHHC22 |
| ENSG00000179988 | PSTK |
| ENSG00000181481 | RNF135 |
| ENSG00000182450 | KCNK4 |
| ENSG00000227372 | TP73-AS1 |
